# Primary Dumbbell-Shaped Lymphoma of the Thoracic Spine: A Case Report

**DOI:** 10.1155/2012/647682

**Published:** 2012-11-28

**Authors:** Antonio Meola, Paolo Perrini, Nicola Montemurro, Paolo di Russo, Giacomo Tiezzi

**Affiliations:** Neurosurgical Department, University of Pisa, Pisa 56100, Italy

## Abstract

Primary spinal non-Hodgkin's lymphoma is extremely rare, and the occurrence of spinal dumbbell-shaped lymphoma is exceptional. We present a case of primary spinal dumbbell-shaped lymphoma to clarify the diagnosis and the management of these lesions. A 45-year-old man presented with sensory symptoms for 8 months. Magnetic resonance imaging of the thoracic spine demonstrated a dumbbell-shaped lesion at the D4–D6 level with spinal cord compression and right foraminal extension at D4–D5 level. The patient underwent D4–D6 laminectomy, with a subtotal resection of the mass. Diffuse large B-cell lymphoma was diagnosed in the pathological examination. He underwent local spinal radiotherapy and chemotherapy. Follow-up evaluation at one year demonstrated no evidence of relapse. Although highly unusual, lymphoma should be included in the differential diagnosis for spinal dumbbell-shaped tumours. After surgery and adjuvant therapy a long-term clinical and neuroradiological followup is mandatory.

## 1. Introduction

Spinal cord compression during the course of non-Hodgkin's lymphoma (NHL) is well described in the literature and occurs in 0.1 to 10.2% of patients [1]. In fact, spinal involvement tends to develop when dissemination has occurred. Primary lymphoma limited to the spinal epidural space is very uncommon and comprises only 6.6% of all intraspinal lymphomas [2]. To our knowledge, only three cases of primary spinal dumbbell-shaped NHL have been described [3–5]. Here we report a case of a 45-year-old man with a primary spinal dumbbell-shaped NHL.

## 2. Case Presentation

### 2.1. History and Examination

**Figure 1:**
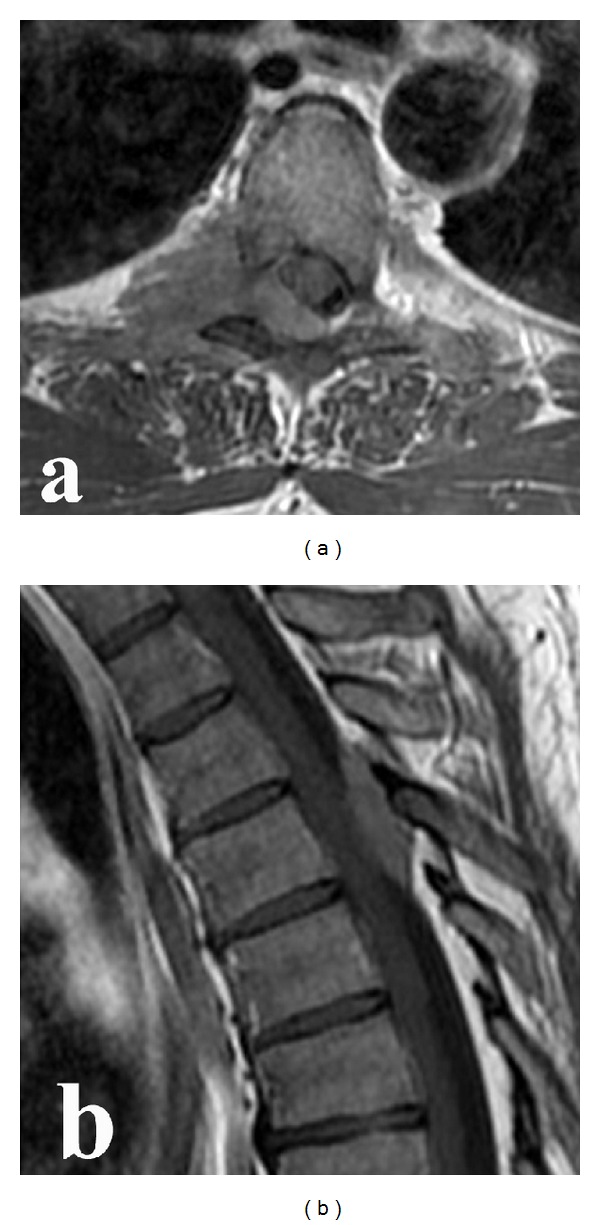
Preoperative axial (a) and sagittal (b) T1-weighted magnetic resonance imaging. Preoperative contrast enhanced magnetic resonance imaging scans showing a homogeneous enhanced dumbbell-shaped intra-extra-spinal mass at the D4–D6 level.

**Figure 2:**
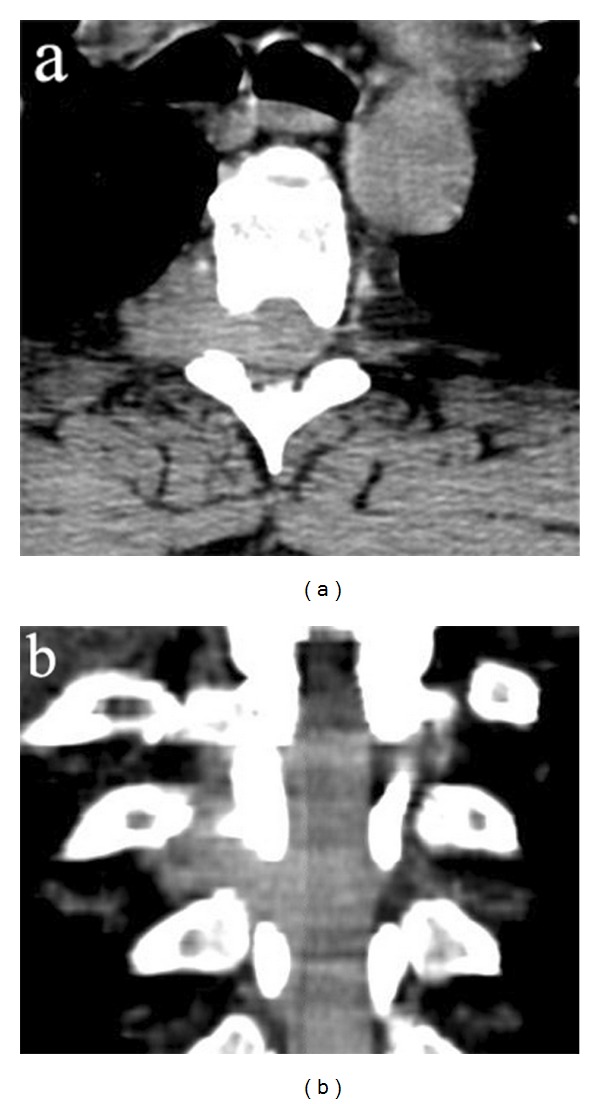
Preoperative axial (a) and coronal (b) computed tomography scans. Computed tomography scans revealing a dumbbell-shaped intra-extra-spinal mass at the D4–D6 level.

A 45-year-old man presented with thoracic back pain since 8 months. The medical history indicated no abnormalities. At the initial clinical examination, the neurological status was normal. Computed tomographic (CT) scan and magnetic resonance (MR) imaging demonstrated a dumbbell-shaped enhancing lesion at the D4–D6 level compressing the spinal cord (Figures 1 and 2). The mass extended out the right D4-D5 foramen to involve the paraspinal muscles. The mass presented homogeneous signal intensity and demonstrated mild enhancement with gadolinium. The supposed diagnosis was nerve sheath tumor.

### 2.2. Operation

The patient underwent a D4–D6 laminectomy with the removal of a soft grayish epidural mass, suggestive for epidural lymphoma. The paraspinal component was subtotally resected.

### 2.3. Pathological Examination

**Figure 3:**
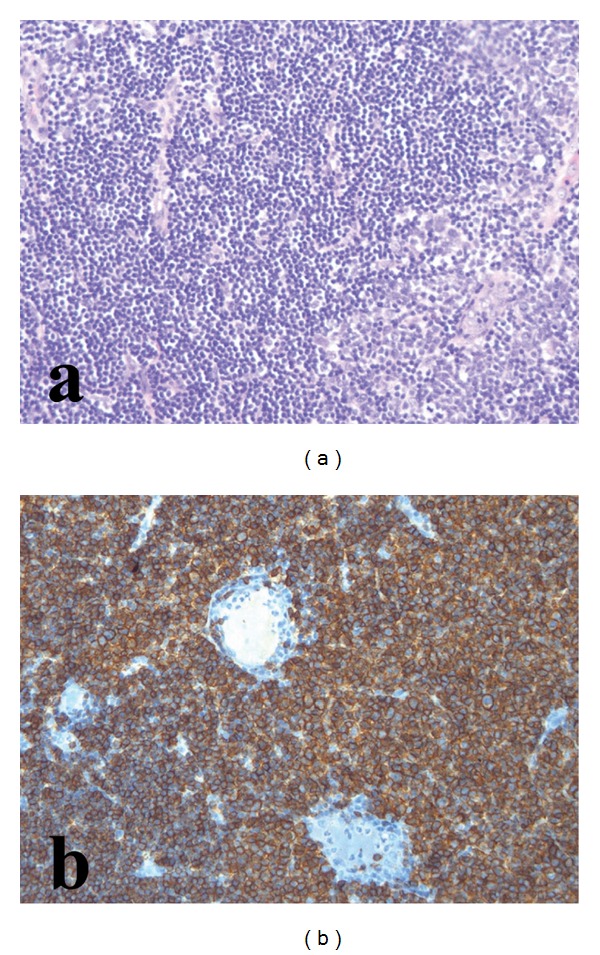
Histopathology of primary dumbbell-shaped lymphoma. Photomicrographs of the pathological specimen showing diffuse large B-cell lymphoma. (a) Sheets of pleomorphic blasts consistent with high-grade lymphoma. H & H, original magnification ×50. (b) Membrane positivity for CD20 confirming B-lymphocytic differentiation. Original magnification ×50.

Light microscopy revealed diffuse large B-cell lymphoma with follicular G3 areas (Figure 3). The immunohistochemical profile was as follows: CD20 and Bcl2 intensely and diffusely positive, CD3 negative, CD10 and Bcl6 positive, CD5 and Cyclin D1-negative with rare scattered positive cells, and CD23 focally positive. The Ki-67 labeling index was >50%. 

### 2.4. Postoperative Course

**Figure 4:**
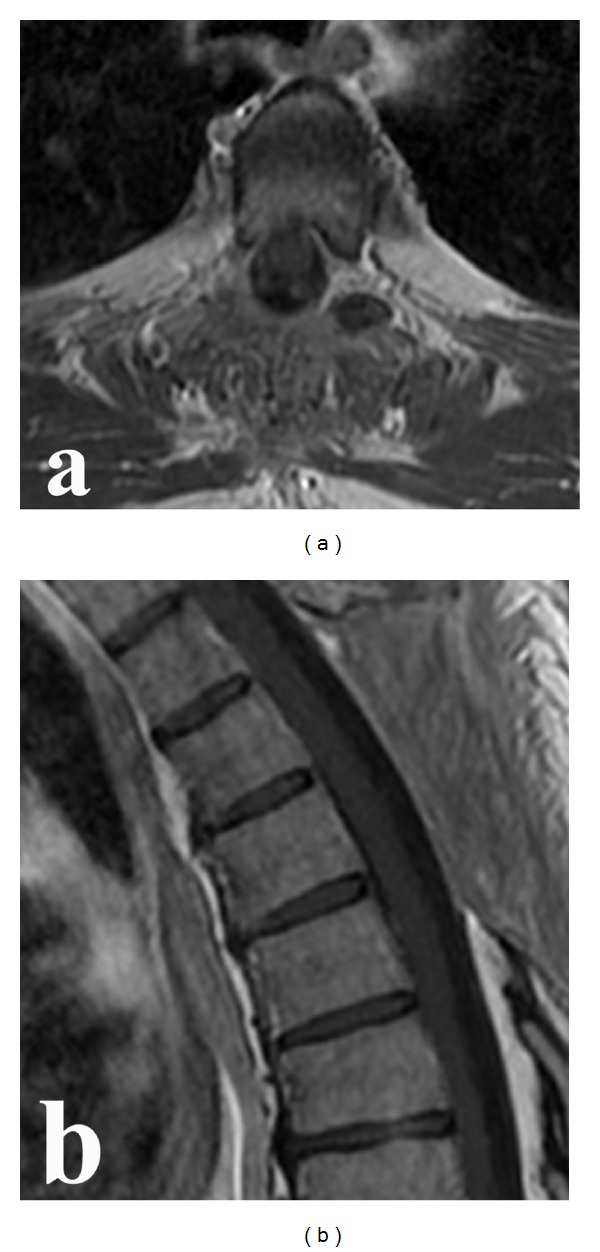
Followup axial (a) and sagittal (b) T1-weighted magnetic resonance imaging. Postoperative contrast enhanced magnetic resonance imaging scans obtained one year after the operation, local radiotherapy, and chemotherapy demonstrated no evidence of relapse.

The postoperative course was uneventful. The staging evaluation revealed no systemic disease. The patient was treated with local spinal radiotherapy and chemotherapy (CHOP-14 for six cycles). At the last followup, one year after surgery, the patient was neurologically intact and restaging performed at this time demonstrated no evidence of relapse (Figure 4).

## 3. Discussion 

The term “dumbbell-shaped tumor” traditionally refers to tumors with a combination of intraspinal and paravertebral involvements showing an hour glass shape. This term has broadened with time to include tumors connecting two or more separate anatomical regions such as intradural, extradural, and paravertebral spaces [6]. The dumbbell-shaped tumors are not uncommon with a reported incidence in the literature of 13,7–17,5% of spinal tumors [6].

Dumbbell tumors include neurogenic neoplasms (schwannoma, neurofibroma, ganglioneuroma, and neuroblastoma), nonneurogenic tumors (meningiomas and sarcomas), and hematopoietic neoplasms (lymphoma or solid leukemic infiltrates). Although this wide histopathological variety, 86% of dumbbell-shaped tumors are schwannomas (69%), neurofibromas (12%) and meningiomas (5%) [6].

Primary spinal dumbbell-shaped NHLs are extremely rare, and including our report four reported cases documented an extraforaminal component associated with an epidural lymphoma [3–5]. Three cases [3, 5] occurred in adults with a mean age of 63 years (range 45–72 yr), and one case occurred in a paediatric patient [4] (11-year-old girl). The location of the tumor was thoracic in two cases; cervical and lumbar in one case, respectively. Thoracic wall pain over a period of months was the clinical presentation in two cases. Progressive weakness was the main complaint in two patients. Histologically, two tumors were diffuse large B-cell lymphoma [4]: one tumor was diffuse follicle lymphoma [5] and one was a mantle cell lymphoma [3].

Preoperative diagnosis of spinal dumbbell-shaped lymphomas is difficult and requires a high index of suspicion. In fact, on the basis of preoperative MRI, two of the four patients were thought to have a nerve sheath tumor. MR imaging features in primary lymphomas of the CNS include a hypointense appearance on T1-weighted images, a hyperintense appearance on T2-weighted images, and homogeneous enhancement after the administration of contrast material [7]. However, these signal characteristics are similar to those of other spinal tumors, such as schwannomas and meningiomas. Conversely, the occurrence of paraspinal infiltration along the muscle fascicles, as in the present case, suggests the possibility of lymphoma and provides a clue to the diagnosis. Since lymphomas are extremely chemo-and radiosensitive, the main goals of surgery are spinal cord decompression and tissue diagnosis. Interestingly, no difference in outcome has been reported between patients undergoing surgery and radiotherapy and patients receiving spinal radiation only [8]. These results support our policy to decompress the spinal cord and not to aggressively resect the paraspinal extension of the lymphoma.

Although highly unusual, lymphoma should be included in the differential diagnosis for spinal dumbbell-shaped tumours. Soft tissues infiltration in the paraspinal space provides a clue for preoperative diagnosis. After surgery and adjuvant therapies a long-term clinical and neuroradiological followup is mandatory.
